# Positive Emotions from Brain Injury: The Emergence of Mirth and Happiness

**DOI:** 10.1155/2020/5702578

**Published:** 2020-01-28

**Authors:** Mario F. Mendez, Leila Parand

**Affiliations:** ^1^Department of Neurology, USA; ^2^Department of Psychiatry and Behavioral Sciences, David Geffen School of Medicine, University of California Los Angeles (UCLA), USA; ^3^Neurology Service, Neurobehavior Unit, V.A. Greater Los Angeles Healthcare System, USA

## Abstract

Brain injury can result in an increase in positive emotions. We describe a 63-year-old man who presented with a prominent personality change after a gunshot wound to the head. He became “content,” light-hearted, and prone to joking and punning. Prior to his brain injury, he suffered from frequent depression and suicidal ideation, which subsequently resolved. Examination showed a large right calvarial defect and right facial weakness, along with memory impairment and variable executive functions. Further testing was notable for excellent performance on joke comprehension, good facial emotional recognition, adequate Theory of Mind, and elevated happiness. Neuroimaging revealed loss of much of the right frontal and right anterior lobes and left orbitofrontal injury. This patient, and the literature, suggests that frontal predominant injury can facilitate the emergence of mirth along with a sense of increased happiness possibly from disinhibited activation of the subcortical reward/pleasure centers of the ventral striatal limbic area.

## 1. Introduction

Positive emotions, such as mirth and happiness, can emerge from selective injury to frontal regions of the brain. The experience of mirth involves cognitive mechanisms for creating and perceiving humor and for responding to it with amusement [1]. Although humor tends to elicit laughter, the processes for humor are distinct from the ability to control laughter, which is disturbed in the condition of pseudobulbar affect [2, 3]. The experience of happiness, on the other hand, includes feelings of bliss, contentment, and euphoric states. Although patients with injuries in the frontal lobes of the brain are often unconcerned and emotional disengaged, they do not usually spontaneously proclaim that they are very happy and content.

We present a patient with a prominent, persistent personality change after a gunshot wound to the head. The patient had Witzelsucht, or excessive and often inappropriate joking and facetious humor [4, 5], along with a continued, light-hearted and happy affect. Witzelsucht has resulted from lesions involving the frontal lobe, particularly the right orbitofrontal region [6]. In addition, the patient had a heightened sense of contentment that appeared to go beyond any disinhibited humor.

## 2. Case Report

A 63-year-old right-handed man with frontotemporal brain injury and a large craniotomy defect was evaluated for noncompliance with medications. The patient had spent 23 years in prison for attempted murder after hearing voices that instructed him to commit suicide. When the police arrived, he began shooting, and they shot him several times including in the head. The patient recovered but was left with a major personality change characterized by persistent feelings of mirth and “happiness.” He had previously suffered from frequent depression and suicidal ideation, which resolved after his head injury such that he never required further treatment or intervention. His medical history for seizures treated with divalproex and lacosamide and right eye blindness was consequent to his head injury. Before being incarcerated, the patient was a college-educated minister, now describing himself as a “blabtist, not a Baptist, because he likes to blab.”

On examination, he was observed to be unconcerned, frequently jesting, punning, or making light-hearted teasing comments to others, and generally not taking his situation seriously. He would occasionally Valsalva to self-inflate his craniotomy defect in order to surprise and amuse anyone around him. His jokes were silly and puerile. For example, he would often ask: “Do want to hear a dirty joke?” After a brief pause, he would announce the punchline as “A white horse fell in the mud” and burst out laughing. When asked, he was readily able to suppress his joking and merriment for prolonged periods without signs of anxiety or distress. He was alert and mostly cooperative with good social reciprocity, eye contact, and turn-taking. His speech was easily interrupted, although he was somewhat tangential, tending to return to an amusing or teasing theme. Otherwise, his speech was normal in rate, prosody, and grammar. His mood appeared excessively good for the context, and his affect was congruent with mood but not overtly euphoric. He did not seem to be aware of the severity of his memory impairment, but he was aware that he had significant cognitive problems.

He underwent a Montreal Cognitive Assessment (MoCA) and scored 16/30, missing the memory and orientation questions, serial 7's, an abstraction, and verbal fluency (only 8 “F” words) [7]. While doing the MoCA, he playfully drew a mouth with the tongue sticking out in the center of the sample cube, and he would alternately tap on the bed and his head on the “A tapping” task. When given “a type of flower” as a cue to help him with a delayed recall item, he replied, “I am a blooming idiot for not remembering this one,” and then proceeded to laugh.

On the physical and neurological examinations, he had prominent signs of his prior gunshot wound to the head with a large craniotomy defect and depression. He had right eye ptosis and proptosis and right facial paralysis. His speech was not dysarthric, and there was no evidence of pseudobulbar palsy or pseudobulbar affect. Surprisingly, his motor examination was normal, including tone and strength bilateral.

**Figure 1:**
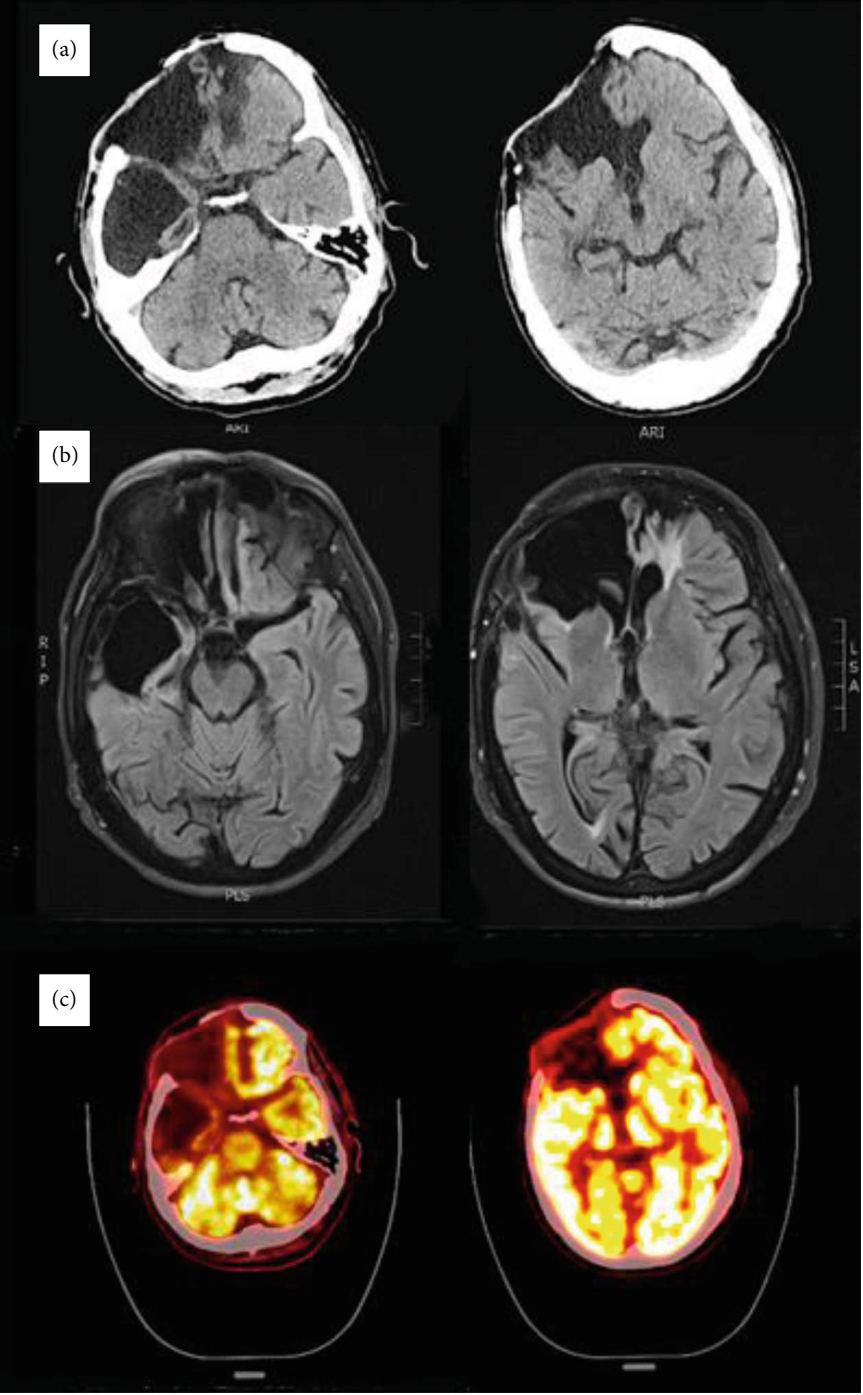
Corresponding axial computerized tomography (CT), magnetic resonance imaging (MRI; fluid-attenuated inversion recovery), and fluorodeoxyglucose positron emission tomography (FDG-PET) scans of the brain. (a) CT shows a large right frontal calvarial vault defect with underlying extensive volume loss/encephalomalacia in the right frontal and anterior right temporal lobes with surrounding gliosis. There is also volume loss and gliosis at the anteroinferior aspect of the left frontal lobe. (b) MRI corroborates the CT findings in a greater detail. (c) FDG-PET shows hypometabolism of right frontal, anterior right temporal, and left anterior inferior frontal lobes corresponding to the CT and MRI findings.

Computerized tomography and magnetic resonance imaging (MRI) of the head showed extensive volume loss and gliosis of the right frontal lobe, the right anterior temporal lobe, and the left orbitofrontal region (see Figure 1). Fluorodeoxyglucose- (FDG-) positron emission tomography imaging showed corresponding hypometabolism in the same areas of the brain without extension to other areas.

He underwent neuropsychological testing which indicated a profound impairment in memory and variable executive functions (see Table 1) [8]. His estimated premorbid full-scale IQ was 115. He was amnestic in both verbal and nonverbal modalities. He had deficits on some executive function tests (verbal and visual fluency tasks), and the most salient executive deficits were in inhibitory control (Stroop Color-Word Interference), with weak switching/response alteration [8]. However, he showed the ability to make verbally reasoned judgments, and he performed well on problem solving/abstraction tasks (e.g., Wisconsin Card Sort Test) [8]. Although he perseverated on some jokes or stories, he did not do so to the same degree on cognitive testing.

The patient underwent examinations with the following specific behavioral tasks:

1. The “Joke and Story Completion Test” [9]. This is a 16-item multiple choice (4 choices) test of humor appreciation. The patient was able to quickly and correctly identify the punchlines of all 16 jokes. He reported them as feeling funny and laughed heartily when he came upon and discovered the correct punchlines from the 4 choices. Additional testing with other jokes confirmed the same ability to identify the funny line and to experience it as funny (laughter)
2. Recognition of the six Ekman and Friesen facial emotions [10]. The patient was shown pictures of people demonstrating the emotions and asked about their facial expressions. His responses were as follows: (1) surprise—“surprised”; (2) anger—“that is murder; a face that ‘I am going to get you'”; (3) disgust—“sadly disgust”; (4) happy—“someone told him a funny joke and his wife is not around”; (5) sad—“Sad face, do I have to hang around anymore for this”; and (6) fear—“Is that animal going to bite me?”
3. The “Sally and Anne” Task and the Hinting Task for screening of Theory of Mind [11]. The patient was given the standard Sally and Anne scenario where Sally brings a doll that Anne moves, unseen by Sally, into a different location. The patient was able to quickly and correctly indicate where Sally would look for her doll. Although originally designed for use in schizophrenia, the Hinting Task has applicability for screening patients with memory impairment. The examiner reads (with rereading as necessary) 10 brief vignettes that consist of two characters, one of whom drops a very clear hint. The patient must indicate what the character really meant (with a subsequent hint as necessary) with scores from 0-2. The patient scored 16/20 (normal 18.03 ± 1.39) [11]
4. The Subjective Happiness Scale (SHS) [12]. The SHS consists of four items, which are scored on a 7-point Likert scale, asking the respondent level of happiness, how the respondent's happiness compares with most of his/her peers, the extent of enjoying life regardless of circumstances, and whether the respondent seems as happy as he might be. The patient's score was 28/28. The patient's elaborations on the questions included “I am content,” “I need some weight to put in my shoes to keep from floating,” and “You will never find me in a miserable state”

## 3. Discussion

This patient had a heightened sense of mirth and happiness after his brain injury. The loss of much of his right frontal and right anterior temporal lobes, and damage to left orbitofrontal cortex, altered his personality towards not just silly joking consistent with Witzelsucht but an actual increase in his appreciation of humor. He also maintained a very positive outlook and increased apparent happiness or contentment, per his report. On examination, he was able to detect jokes and identify them as funny, and he consistently described himself as very happy despite his brain injury and situation.

Beyond his joking or “Witzelsucht” from the German words for joke (Witz) and addiction (Sucht) [4, 5], this patient had increased mirth. Mundane experiences and others' jokes caused him amusement. Investigators have characterized the neurobiology of humor as involving several modular aspect [1, 13]. The first is the cognitive aspect, or getting the joke, namely, the perception of incongruity or of incompatibility between an anticipated perspective and the punchline. Second, with resolution of the incongruity, there is the actual humor appreciation or mirth involving the dopaminergic pleasure/reward centers of the ventral striatum and nucleus accumbens (VS/NA) [14–16]. The frontal lobes participate in incongruity detection and resolution, with the left frontal more responsive to simple humor [16–18], and the right more engaged with complex humor [14, 19–21]. In addition, other regions may contribute to incongruity detection and resolution, such as the temporoparietal junction, the precuneus, the posterior cingulate cortex, and the parahippocampal gyrus [22]. Once incongruity is resolved, the new explanation triggers emotionally pleasurable responses experienced as mirth [9, 14, 15, 23, 24]. Since this patient could “get a joke,” his changes appeared at the level of the ease of elicitation of mirth.

The regions associated with the experience of mirth include the VS/NA as well as connections from the anterior cingulate gyrus (ACG), posterior insula, and frontal (left>right) lobe [25–28]. Deep brain stimulation of the VS/NA induces mirth and enhances effective connectivity from the ACG to the VS [25, 26]. As well as promoting surprise, electrical stimulation of the rostral pregenual ACG can also elicit laughter with mirth [27, 29, 30]. The experience of mirth can occur with the rerepresentation and integration of interoceptive information in the insula [28]. Finally, the frontal lobes trigger humor appreciation through connections with these structures [14–16, 20, 29, 31, 32].

The formation and regulation of happiness seem to be associated with significant reductions in activity in the right prefrontal cortex, as well as increased activity in the VS/NA [33]. The left frontal lobe may produce a default state biased towards happy or positive interpretations [34]. For example, cortical sites that produce mirth when stimulated tend to be located in the dominant hemisphere close to language areas [35, 36]. Furthermore, disruption of left frontostriatal emotion regulation systems can impair the ability to suppress positive emotions such as happiness [37]. Together, these findings, as well as the patient's increased appreciation of humor, suggest that his brain lesion facilitated or released his VS/NA area from any negative input or inhibition. This view must be interpreted cautiously from the analysis of a single patient. There may be other explanations for the patient's positive emotions, such as the simple relief from depression after his head injury, or as a result of alleviation of stress from no longer functioning as a minister. Nevertheless, his personality change was quite dramatic shortly after recovering from his gunshot wound to the head.

We conclude that positive emotions such as mirth and happiness can emerge from brain lesions and persist. The loss of much of the right frontal and right anterior temporal lobes and damage to the left orbitofrontal cortex facilitated a positive sense of amusement and a positive outlook described as “contentment” by this patient. The literature suggests that this can occur in patients with predominant damage to the right frontal lobe, but also affecting left frontostriatal circuits. These observations warrant further investigation as they speak to the source of positive emotions in humans.

## Figures and Tables

**Table 1:** Neuropsychological tests [8].

	Raw score	%ile
Digit span	17	5
Trail making A time (seconds)	58	4
BNT with stimulus cues (0/1)	55/60	24
COWAT verbal fluency (F (8) + A (12) + S (5))	25	4
Category (animal) naming	16	18
WAIS-IV block design	20	9
Hooper visual organization test total	24.5	63
*Memory*		
CVLT (short form) trials 1-4 total	17	3
CVLT (short form) short delay and long delay free recall	3, 0	2
CVLT (short form) long delay cued recall	0	<1
CVLT (short form) total recognition discrimination	1.8	31
WMS-IV logical memory I	21	9
WMS-IV logical memory II	0	0.1
WMS-IV logical memory II recognition	15/23	3-9
BVMT-R trial 1-3	0-3-2	<1
BVMT-R total recall, delayed recall, percent retained	5, 0, 0%	<1
*Executive* (including trail making A, COWAT, and block design)		
Trail making B time (seconds)	106	12
WCST-64 categories completed and trials to complete 1st	4, 11	>16
WCST-64 total errors	15	53
WCST-64 perseverative responses and errors	11, 10	21
WCST-64 nonperseverative errors	5	53
WCST-64 conceptual level responses	46	42
D-KEFS design fluency test filled dots total correct	5	16
D-KEFS design fluency test empty dots total correct	4	9
D-KEFS design fluency test switching total correct	2	5
Sum of scaled scores (7, 6, 5)	18	5
D-KEFS tower test total achievement score	10	9
WAIS-IV similarities	21	25
Stroop color naming (seconds)	80	23
Stroop word reading (seconds)	54	31
Stroop Color-Word Interference (seconds)	214	1
Grooved pegboard dominant (R) time (seconds)	122	2
Grooved pegboard non-dominant (L) time (seconds)	149	2

BNT = Boston Naming Test; BVMT-R = Brief Visuospatial Memory Test-Revised; COWAT = Controlled Oral Word Association Test; CVLT = California Verbal Learning Test; D-KEFS = Delis-Kaplan Executive Function System; Sec = seconds; WAIS-IV=Wechsler Adult Intelligence Scale-IV; WCST-64 = Wisconsin Card Sorting Test 64 Card Version; WMS-IV=Wechsler Memory Scale-IV.
